# GLP-1 Receptor Agonists in Aesthetic Surgery: An Expert Video Roundtable Discussion

**DOI:** 10.1093/asjof/ojag152

**Published:** 2026-07-29

**Authors:** Ryan E Austin, Daniel J Gould, Renee Burke, Kye Higdon

The authors are pleased to present an expert roundtable discussion on GLP-1 receptor agonists in aesthetic surgery (Video). The discussants include a panel of leading experts in aesthetic surgery, and the video roundtable was recorded live at The Aesthetic MEET 2026 in Boston, MA. The panelists began with a brief overview of the history of GLP-1s and their recent surge in popularity, and the effect this has had on their patients. Many plastic surgeons are now working with patients who they may have previously turned down for surgery and helping guide these patients to a healthier BMI in preparation for surgery. The discussants agreed that they tend to see more skin laxity with patients on GLP-1s, and they are using more soft tissue support than they did previously. They stressed the importance of a personalized approach to postoperative care for these patients, which can include a high-protein diet and careful management of their other medications. They also noted the wide-ranging effects of GLP-1s and the importance of taking a holistic approach to care, considering the patient's nutrition, mental health, and expectations for the procedure. The discussants concluded by agreeing that the world of GLP-1s and peptides is just beginning and that these drugs will have a significant presence in aesthetic surgery going forward.

## Supplementary Material

ojag152_Supplementary_Data

